# Metabolic limitations of soil microorganisms during the decay of *Salix psammophila* sand barriers

**DOI:** 10.3389/fmicb.2025.1585493

**Published:** 2025-06-03

**Authors:** Yumei Liang, Xiaoting Duan, Xin Guo, Ruiting Jia, Qi Tian

**Affiliations:** ^1^College of Desert Control Science and Engineering, Inner Mongolia Agricultural University, Hohhot, China; ^2^Inner Mongolia Key Laboratory of Desert Ecological System, Inner Mongolia Academy of Forestry Sciences, Hohhot, China; ^3^Water Resources Bureau of Dalat Banner, Ordos, China; ^4^Natural Resources Bureau of Ertokqian Banner, Ordos, China; ^5^College of Computer Science, Inner Mongolia University, Hohhot, China

**Keywords:** *Salix psammophila* sand barriers, soil enzyme stoichiometry, soil physical and chemical properties, nutrient limitation, desertification control

## Abstract

*Salix psammophila* sand barriers are the main measure used in desertification control engineering technology, which is widely used in China’s northwest desert region. To clarify the change characteristics and driving factors of soil enzyme stoichiometric ratios and microbial metabolic limitation during the decay of *S. psammophila* sand barriers, we determined soil basic physical and chemical properties and C:N:P stoichiometric ratios in the sand-buried portion of the *S. psammophila* sand barriers from 1 to 10 years. The results showed that (1) soil C:N showed an increasing trend, but soil N:P showed a decreasing trend over time. The activities of leucine aminopeptidase, β-1,4-n-acetylglucoside, and alkaline phosphatase first increased and then decreased with the increase of *S. psammophila* sand barrier years, and reached the maximum value at 6 years. (2) Redundancy analysis revealed that soil stoichiometric ratios were the main factors driving soil enzyme activities and their stoichiometry. (3) The soil enzyme C:N:P stoichiometric ratio was approximately 0.8:1:1. The enzyme vector lengths ranged from 0.66 to 1.09, and the vector angles ranged from 41.86° to 49.70°. Soil microorganisms were limited by nitrogen in the early stages (<5 years), while in the later stages (5–10 years) they were phosphorus-limited. Therefore, in the process of *S. psammophila* sand barriers assisting in the restoration of vegetation, it is considered to add an appropriate amount of nitrogen fertilizer to the soil in the first 5 years and add a small amount of phosphate fertilizer in the last 5 years to ensure ecosystem stability. Our findings are of great significance for artificial interventions for vegetation restoration and desert ecological conservation in desert areas.

## 1 Introduction

As the main sandy shrub native species in the Kubuqi Desert, *S. psammophila* has strong drought and high-temperature tolerance and sprouting ability, which makes it a good material for sand barriers. Usually, the stem of *S. psammophila* is cut into a length of 50–60 cm, half of it is inserted into the sand soil, and half is exposed to the atmosphere, and laid on the surface of the dune according to different specifications (e.g., 1 m × 1 m or 1.5 m × 1.5 m, etc.) to change the speed, direction, and structure of the wind-sand flow (Gao et al., 2013). Compared with some traditional wind-tight or tightly structured sand barriers, such as clay, pebbles, etc., the *S. psammophila* sand barrier is a wind-transparent structure with a more significant protective effect, and it has the dual functions of plant measures and engineering measures. Therefore, it is one of the important measures for artificial intervention of desertification control means in the arid region of Northwest China. Many previous studies have shown that it can effectively reduce near-surface wind speed, increase surface roughness, improve soil fine particulate matter and soil organic matter, and promote vegetation recovery (Gao et al., 2013; Zhang et al., 2016; Dai et al., 2019).

However, the lower part of the *S. psammophila* sand barriers, due to long-term burial in a sandy environment, is subjected to the phenomenon of decay and disintegration by the irregular hygroscopic-desorption of soil moisture and decomposition by microorganisms (Wang et al., 2021a). Inevitable natural degradation releases nutrient elements C, P, and K from the sand barriers to the soil in a fluctuating state (Wang et al., 2021b). In this process, the community structure and diversity of soil microorganisms are also directly affected (Liang et al., 2021; Liang et al., 2023). Soil microorganisms play an important role in soil organic matter decomposition and nutrient transformation (Shi et al., 2020), and they secrete extracellular enzymes according to changes in soil nutrients, thus obtaining carbon (C), nitrogen (N), and phosphorus (P) from the soil to meet their own growth needs. This process is a catalyst and key driver of soil nutrient cycling and material transformation (Cui et al., 2020; Hill et al., 2014). The absorption and utilization of nutrients such as C, N, and P in soil are regulated by soil enzymes in the environment (Wang et al., 2015). It is generally believed that β-1,4-glucosidase (BG), alkaline phosphatase (ALP), β-1,4-N-acetylglucosaminidase (NAG), and leucine aminopeptidase (LAP) can catalyze the production of bioavailable terminal monomers to regulate C, N, and P in soil (Sinsabaugh et al., 2008). These enzymes’ activities can serve as proxy indicators of microbial resource allocation for C, N, and P acquisition (Schimel and Weintraub, 2003; Fanin et al., 2016).

The ratio of microbial extracellular enzymes can reflect the biochemical balance between microbial metabolism and nutritional requirements (Sun et al., 2021), and is often used to measure the nutrient requirements and limitations of soil microorganisms (Hill et al., 2010). Sinsabaugh et al. (2010) put forward the concept of soil enzyme stoichiometry and found that the stoichiometric ratio of soil C:N:P enzyme activity on the global scale, that is, ln BG: ln(NAG + LAP): ln ALP, was 1:1:1 (Zhong et al., 2021). However, the expression of soil microbial enzymes will vary with changes in the soil environment, and soil enzymatic ratios may no longer follow a 1:1:1 relationship. The C:N:P ratio of enzyme activities may be influenced by climate, vegetation, soil properties, and human activities (Xu et al., 2017). Existing studies have not concluded that soil microbial enzyme activity is significantly affected by a certain factor, and most findings vary by study area. For example, Mo et al. (2020) found that soil enzyme activity was significantly associated with several soil physicochemical properties. Li et al. (2021) found that total potassium was a key factor affecting soil enzyme activity in a study of typical farmland. Tan et al. (2021) also reported that soil enzyme activity was mainly nutrient-driven. However, the relationship between soil enzyme activities and nutrient changes during the decay of *S. psammophila* sand barriers is not known.

In recent years, research on *S. psammophila* sand barriers has focused on the physicochemical properties of sand barrier degradation under UV irradiation (Wang et al., 2024), soil physicochemical properties, changes in microorganisms, and shifts in biomass in the decay process of sand barriers (Liang et al., 2021; Liang et al., 2023). Changes in soil enzyme stoichiometric ratio and microbial metabolic restriction during the decay of the *S. psammophila* sand barrier are still poorly understood. Therefore, in this paper, the soil around the sand *S. psammophila* sand barriers in different years of the Kubuqi Desert was selected as the research object to investigate the change of soil enzyme activities and the nutrient limitation of microorganisms. We make the following assumptions: (1) The decay process of *S. psammophila* sand barriers may promote an increase in soil enzyme activities and a responsive relationship with soil properties. (2) With the increase of sand barrier decay, soil enzyme C:N:P stoichiometry may change, and microbial metabolism is limited by one or more nutrients. This study was to further clarify the characteristics and driving factors of soil enzyme activities and enzyme stoichiometry in the process of *S. psammophila* sand barrier decay to provide a scientific basis for the restoration of desert ecosystems and the management of soil nutrients.

## 2 Materials and methods

### 2.1 Study area

**Figure 1:**
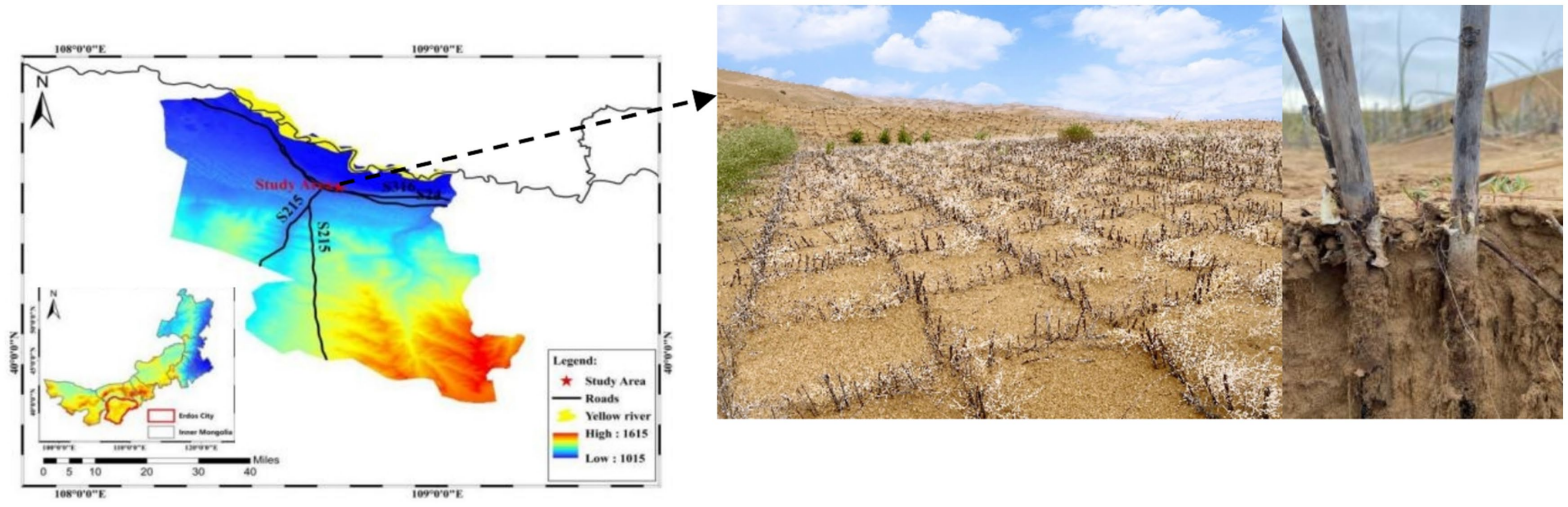
Location of the study area (Hangjin County, Ordos, Inner Mongolia, China).

The study area is located in the Kubuqi Desert in Duguitala Town, Ordos, Inner Mongolia (Figure 1). This region is located in the north of the ridge line of the Ordos Plateau (40°29′16″–40°29′35″N, 108°40′09″–108°41′21″E) and has a temperate continental arid climate with a great temperature difference between seasons, abundant sunshine, and a short frost-free period. The average temperature in January is −13.4°C, the average temperature in July is 22.8°C, the average annual precipitation is 311.8 mm, and the annual average wind speed is 2.8 m/s. Sandy soils are dominated by medium and fine sands with low clay and powder granules. Landform types include mobile dunes, fixed dunes, and semi-fixed dunes. Vegetation types are primarily *Salix psammophila*, *Agriophyllum squarrosum*, *Psammochloa villosa*, *Corispermum hyssopifolium*, and *Artemisia ordosica*. To ensure the normal operation of the road through the sand, prevent road damage caused by wind erosion or sand burial, and reduce the intrusion of wind and sand flow, semi-concealed *S. psammophila* sand barriers were installed on both sides of the expressway.

### 2.2 Experimental design

In July 2021, we collected samples of *S. psammophila* sand barriers laid on gentle dunes on both sides of the Xingba sand-traversing expressway. In this study, all study sites were located next to a highway through the desert, with flat terrain. These *S. psammophila* sand barriers were installed every year in cooperation with the local forestry department, which was mainly used for wind and sand protection of desert highways and long-term observation of the experiment. Thus, it was possible to accurately determine the age of the sand barriers. Using the method of “space” instead of “time,” the sample plots of 1–10 years were selected for the study. Three 1 m × 1 m grids were randomly selected in each sample area, with an interval between the grids of over 10 m. One sample was taken from the center of each of the four sides of each grid, and the 12 samples taken from each sample plot were mixed homogeneously to form one test sample. There are three replicates of sample plots of the same age. A total of 360 samples were collected. The test samples were collected from the soil in the most serious part of the *S. psammophila* sand barrier decay, and the thickness was 5 cm. We first gently brush the soil off the surface of the sand barrier and collect it into the envelope. A small shovel was then used to collect soil that was tightly packed within 1 cm of the sand barrier. After the sample was mixed evenly, it was placed in a Ziplock bag and transported back to the laboratory in a refrigerated box (4°C). The soil samples were passed through a 2-mm sieve indoors and divided into two parts. One part was placed in a cool place and air-dried for the determination of soil physical and chemical properties, while the other part was placed in a refrigerator at 4°C for the determination of enzyme activities.

### 2.3 Determination of soil physicochemical properties

Soil water content (SWC) was determined using the oven-drying method. The soil pH value (water:soil = 2.5:1) was measured using a PHSJ-4A pH meter (Zhangqiu Meihua International Trading Co., China). The soil organic carbon (SOC) was determined using the Walkley-Black method (Nelson and Sommers, 1996). The soil available nitrogen (AN) was obtained by the continuous alkali hydrolysis reduction diffusing method (Cornfield, 1960). The soil available phosphorus content (AP) was measured by the Olsen method (Olsen and Sommers, 1982). The content of available potassium (AK) in soil was obtained by NH_4_OAc extraction and flame photometry. The soil total nitrogen (TN) was determined using the semi-micro Kjeldahl method (Bremner, 1996), and the determination of soil total phosphorus (TP) was conducted using the sodium hydroxide fusion-molybdenum antimony anti-colorimetric method. Soil C, N, and P stoichiometric ratios use the ratio between SOC, TN, and TP.

### 2.4 Soil enzyme activity assay

Soil extracellular enzyme activity was determined by the 96-microtiter enzyme plate fluorescence assay (Saiya-Cork et al., 2002). For BG, NAG, LAP, and ALP assays, 1 g of fresh soil from a 2 mm sieve was weighed, and 125 mL of distilled water was added and shaken for 2 h (25°C, 180 r/min) to form a suspension. The sample suspension, a substrate solution, and a buffer were injected into a 96-well enzyme standard plate in a specific sequence using a multichannel pipette. After incubation for 4 h at 25°C under light-proof conditions, 50 μL of 0.5 mol L^−1^ NaOH solution was added to each well to terminate the reaction, and 250 μL was transferred to BG, which is closely related to C cycling labeled 96-well plates (excitation wavelength 365 nm, emission wave length 450 nm). The soil enzyme activity was calculated after a negative control and quenching correction. The unit of enzyme activity was nmol g^−1^ h^−1^.

### 2.5 Data analysis

The vector length (Vector L) and angle (Vector A) of enzyme stoichiometry are calculated using Equations 1, 2 (Moorhead et al., 2016):


(1) $$\begin{array}{l} \text{Vector}\;L=\{{\left[\ln(\text{BG}):\ln(\text{NAG}+\text{LAP})\right]}^{2}+\\ \;\;\;\;\;\;\;\;\;\;\;\;\;\;\;\;\;\;\;\;\;\;\;\;\;\;\;{{\left[\ln(\text{BG}):\ln(\text{ALP})\right]}^{2}\}}^{1/2} \end{array}$$

(2) $$\begin{array}{l} \text{Vector}\;A=\text{Degrees}\\ \left\{\text{ATAN}2[\ln(\text{BG}):\ln(\text{ALP}),\ln(\text{BG}):\ln(\text{NAG}+\text{LAP})]\right\} \end{array}$$

In the formula, the length of Vector L indicates the degree of soil microbial C limitation. The size of Vector A indicates the degree of soil microbial N and P limitation; when Vector A deviates from 45°, the soil is N-limited or P-limited, and the greater the upward deviation, the stronger the P restriction, while the greater the downward deviation, the stronger the N limitation. ATAN2 is the arc tangent of the line from the origin to the point [lnBG:ln ALP, and lnBG:ln(NAG + LAP)]. Degrees represent the tangent angle.

The data were tested for normality and homogeneity of variance. Data that had a non-normal distribution and non-homogeneity of variance were log-transformed. One-way analysis of variance was used to analyze differences in the soil physicochemical properties, soil enzyme activity, enzyme stoichiometry ratios, vector length, and vector angle of the *S. psammophila* mechanical sand barriers of different ages. Soil C:N enzyme activity ratios were expressed as ln(BG):ln(NAG + LAP), soil C:P enzyme activity ratios as ln(BG): ln(ALP), and soil N:P enzyme activity ratios as ln(NAG + LAP):ln(NAG + LAP). Vector transformation of enzyme stoichiometry translates biochemical reactions into a computable mathematical framework. Tukey was used for post-hoc multiple comparisons. Canoco 5.0 software was used to complete the redundant relationship analysis between soil physicochemical properties, soil enzyme activity, and enzyme stoichiometric ratio. Origin 2021 and GraphPad Prism 9.4.0 software were used for drawing.

## 3 Results

### 3.1 Physico-chemical properties of *Salix psammophila* sand barrier soil in setting different years

**Figure 2:**
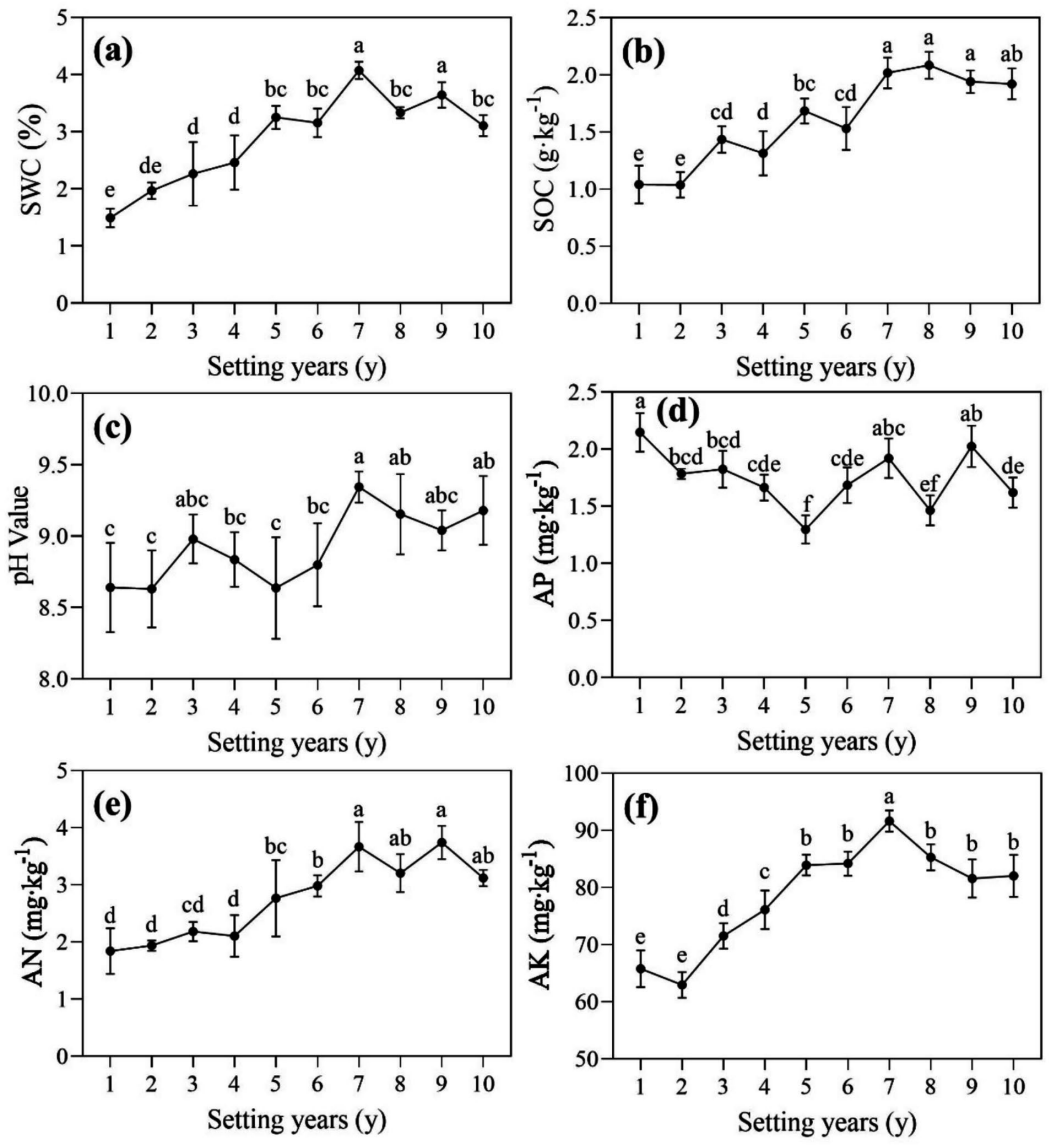
**(a-f)** Physical and chemical properties of soil around *S. psammophila* sand barriers in different years. SWC, soil water content; SOC, soil organic carbon; AP, available phosphorus; AN, available nitrogen; AK, available potassium. Different letters (i.e. a–f, in the figure) indicate significant differences between different setting years (*p* < 0.05).

There were differences in soil properties around the *S. psammophila* sand barrier at different years (Figure 2). SWC and AK were significantly higher than the other groups at 7 years (*p* < 0.05), with values 2.73 and 1.46 times higher than those at 1 and 2 years, respectively (Figures 2a,f). The first 5 years of decaying *S. psammophila* sand barrier can increase the content of AK, but most of them showed no significant difference after 5 years. As shown in Figures 2b,f, SOC and AK generally showed a trend of increasing first and then decreasing, with levels ranging from 1.04 to 2.08 g/kg and 62.95 to 91.62 mg/kg, respectively. AN was highest in the 9-year soil and was 2.03 times higher than in the 1-year soil. The overall pH of the soil was alkaline (8.63–9.34), and with the increasing year of the sand barrier, there was a gradually increasing trend (Figure 2c). In addition, the soil available phosphorus around the 1-year *S. psammophila* sand barrier was significantly higher than that of the other (Figure 2d, *p* < 0.05).

**Figure 3:**
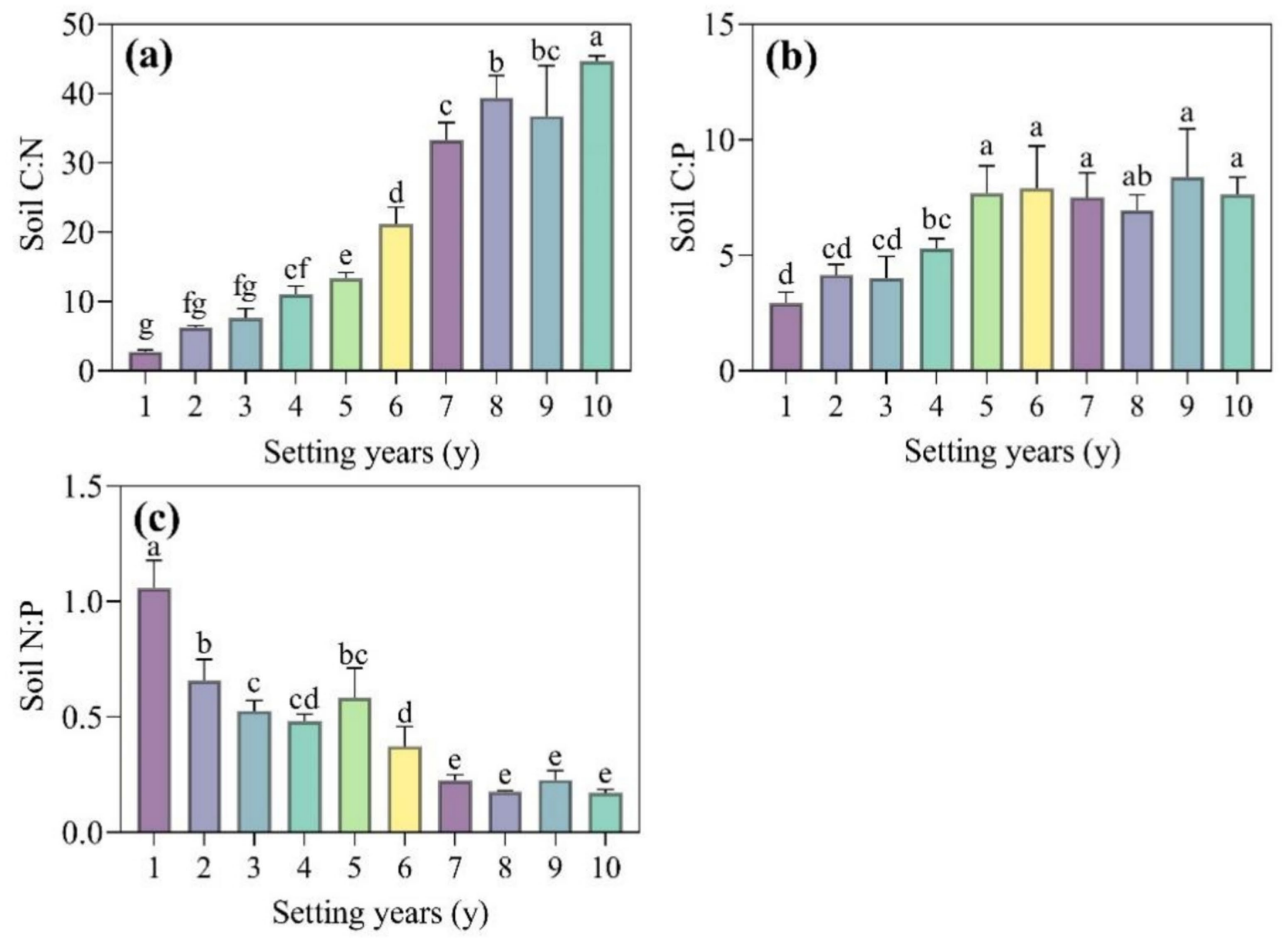
**(a–c)** Soil stoichiometric ratios of *S. psammophila* sand barriers in different years. Different letters (i.e. a–g, in the figure) indicate significant differences between different setting years (*p* < 0.05).

The decay process of *S. psammophila* sand barriers had a significant effect on soil stoichiometric ratios (Figure 3, *p* < 0.05). With the increase of time, soil C:N showed an upward trend and reached the maximum value in 10 years (Figure 3a). Soil C:P also showed an overall increasing trend, but there was no significant difference after 5 years (Figure 3b). Soil N:P showed a decreasing trend with time, but there was no significant difference after 7 years (Figure 3c).

### 3.2 Soil enzyme activity and its stoichiometric characteristics at different setting years

**Figure 4:**
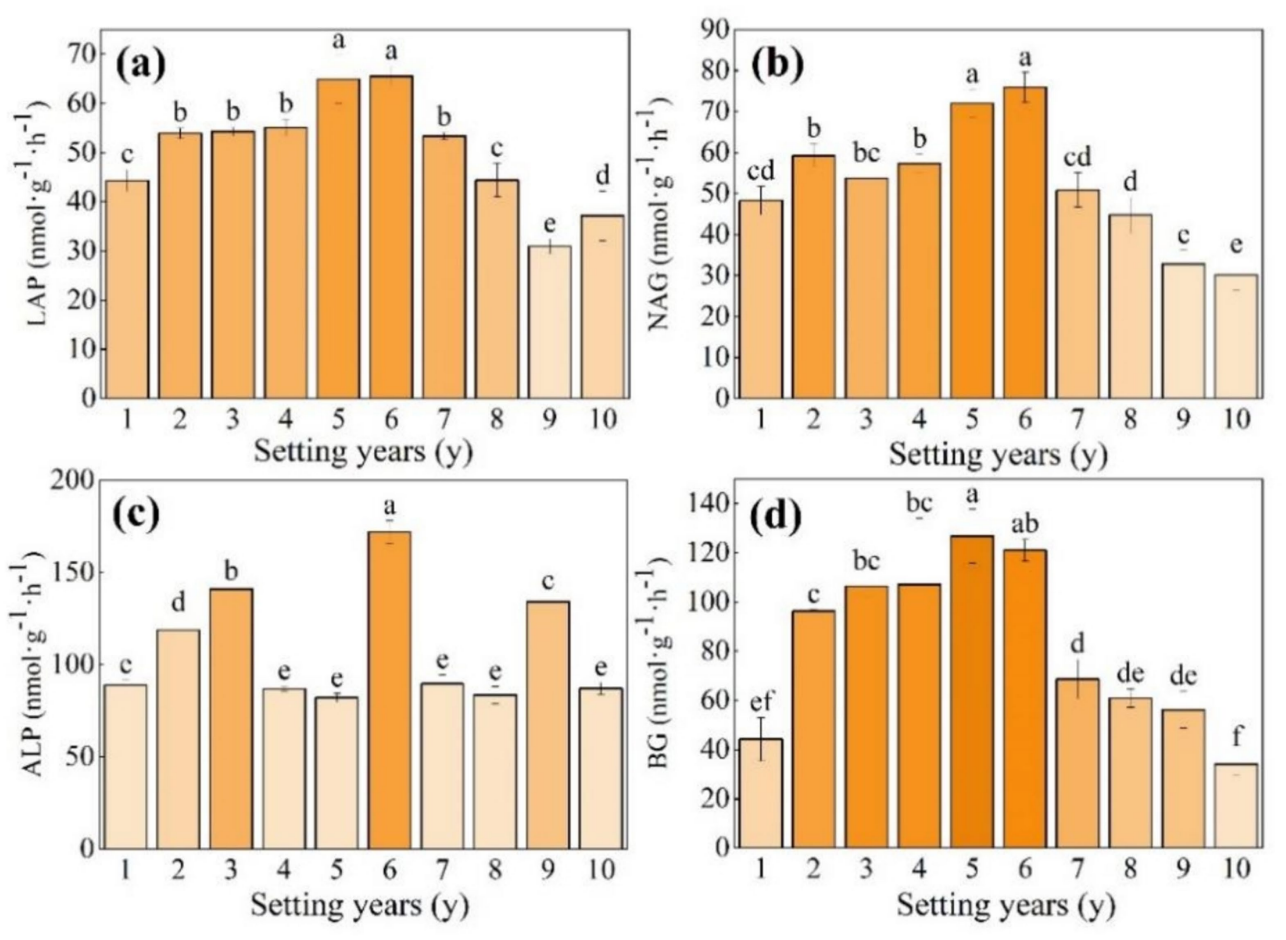
**(a–d)** Soil enzyme activity of the *S. psammophila* sand barrier at different setting years. Different letters (i.e.a–f, in the figure) indicate significant differences between different setting years (*p* < 0.05).

As shown in Figures 4a,b, the activities of LAP and NAG, which are involved in the N cycle, showed a trend of first increasing and then decreasing with time; the ALP activity, which is involved in the P cycle, showed a fluctuating trend of increasing first and then decreasing with time, with a variation range from 82.06 to 171.85 nmol g^−1^ h^−1^ (Figure 4c); and the BG activity, which participates in the C cycle, showed a trend of increasing first and then decreasing with time (Figure 4d). The activity of NAG and LAP was significantly higher than that in other setting years when the *S. psammophila* mechanical sand barrier was set for 5 years and 6 years (*p* < 0.05), at 1.57 and 1.48 times higher compared with that at 1 year. The BG activity reached the maximum value when the sand barrier was set for 5 years, at a 2.86-fold increase compared with the mechanical sand barrier set for only 1 year.

**Figure 5:**
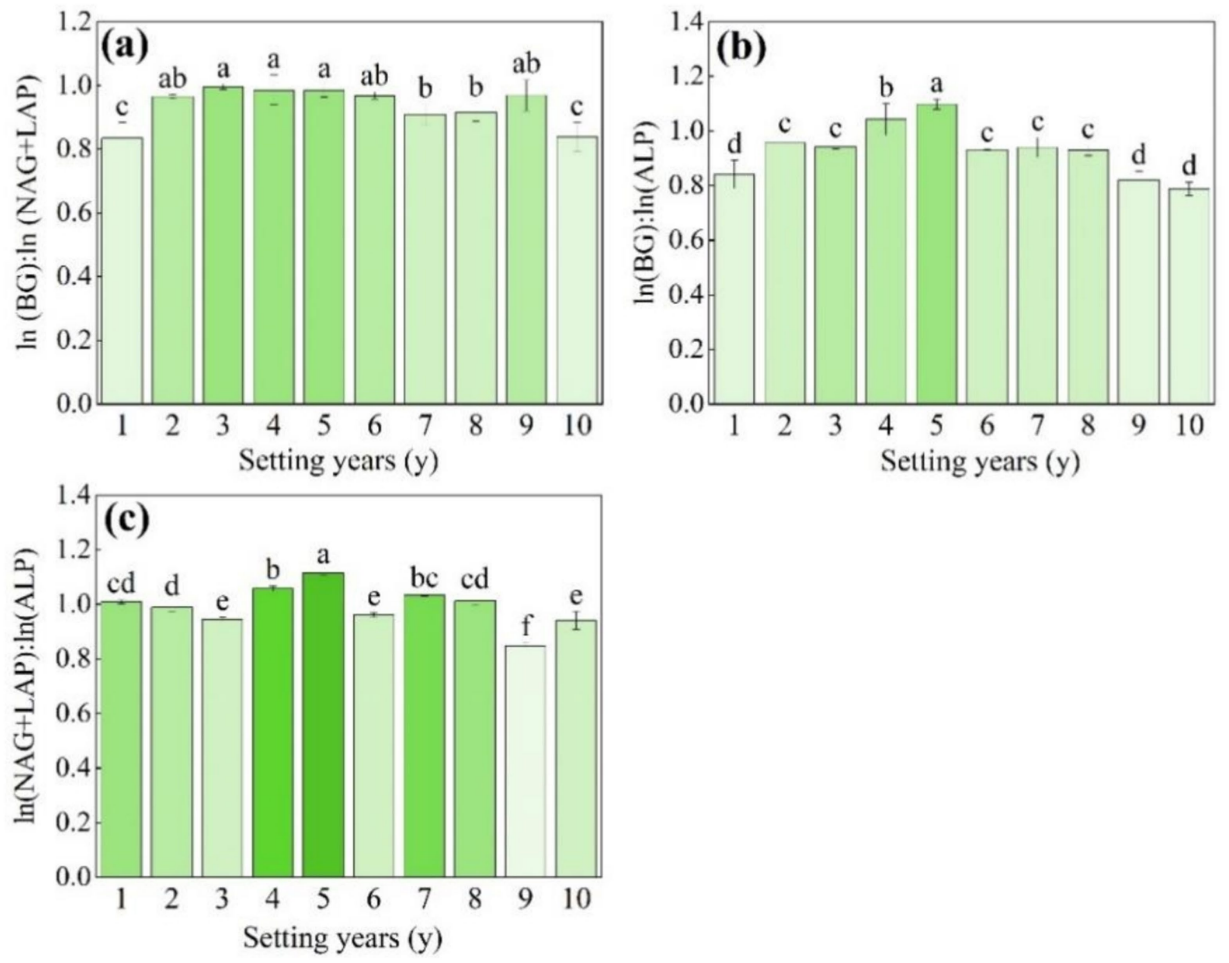
**(a–c)** Soil enzyme stoichiometry ratios of the *S. psammophila* sand barrier at different setting years. Different letters (i.e. a–f etc. in the figure) indicate significant differences between different setting years (*p* < 0.05).

As shown in Figure 5a, the ln(BG):ln(NAG + LAP) (enzyme C:N) varied in the range of 0.8–1.0, and there were no significant changes from 3 to 5 years (*p* > 0.05). The ln(BG):ln(ALP) (enzyme C:P) varied from 0.8 to 1.1, and the activity of soil enzyme C:P was significantly higher than that of other setting years when the *S. psammophila* mechanical sand barrier had been set for 5 years (Figure 5b, *p* < 0.05). The ln(NAG + LAP): ln(ALP) (enzyme N:P) varied in the range of 0.8–1.1, the 5-year soil enzyme N:P was the highest, and there was no significant difference in the 3-year, 6-year, and 10-year soil enzyme N:P (Figure 5c, *p* > 0.05). The mean value of the soil enzyme stoichiometric ratio C:N:P between the *S. psammophila* mechanical sand barriers set for different numbers of years was about 0.8:1:1.

**Figure 6:**
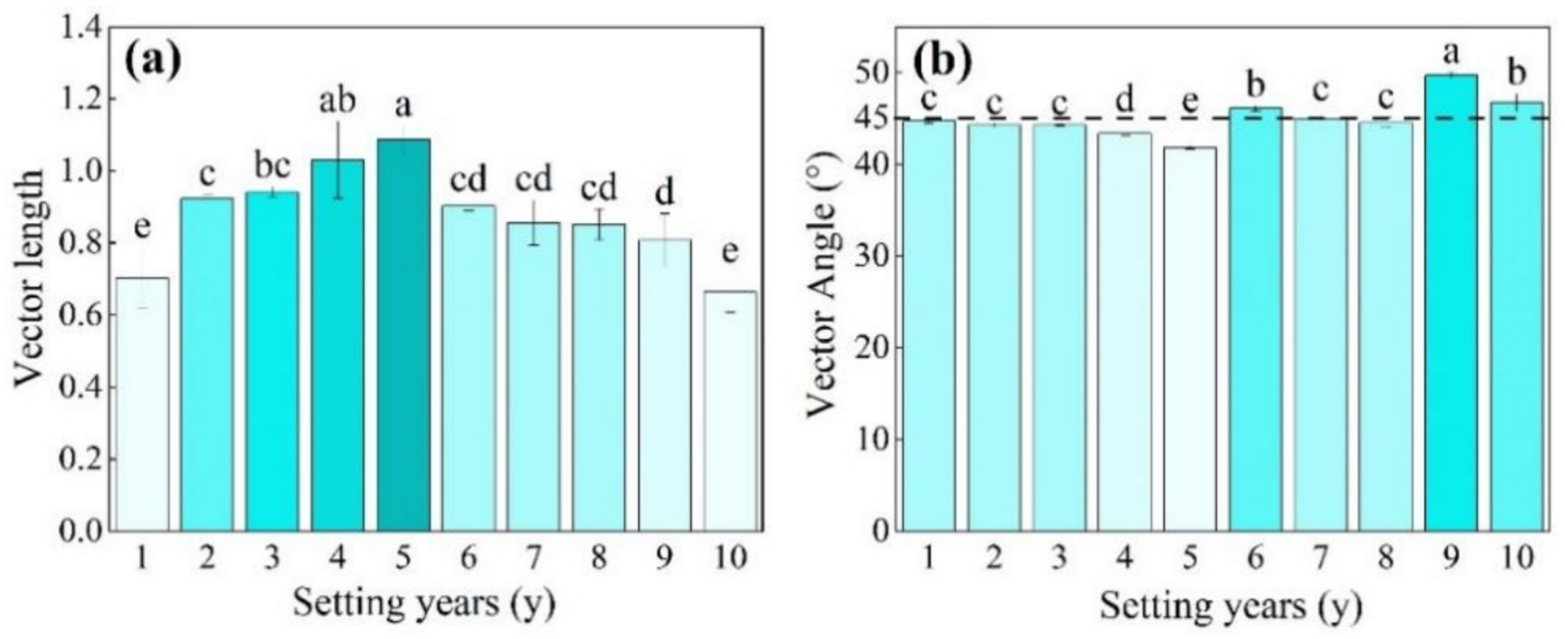
**(a, b)** Length and angle of the soil enzyme vector of the *S. psammophila* sand barrier at different setting years. Different letters (i.e. a–d, in the figure) indicate significant differences between different setting years (*p* < 0.05).

As shown in Figure 6a, when the *S. psammophila* sand barrier was set for 5 years, the length of the soil enzyme vector was significantly higher than that of other setting years (*p* < 0.05), and the Vector L of 1–5 years varied in the range 0.70–1.09. The 6–10-year change tended to be stable at between 0.66 and 0.90. The soil enzyme Vector A was 41.86°–49.70°. Vector A was less than 45° when the sand barriers were set between 1 year and 5 years, and Vector A was greater than 45° between 6 years and 10 years, where the 9-year mechanical sand barrier inter-perimeter soil Vector A was significantly greater compared with the other setting years. The length of the soil enzyme vector of 10-year-old sand barriers decreased significantly (Figure 6b; *p* < 0.05).

### 3.3 Relationship between soil physicochemical properties and soil enzyme activity and their stoichiometric ratios

**Figure 7:**
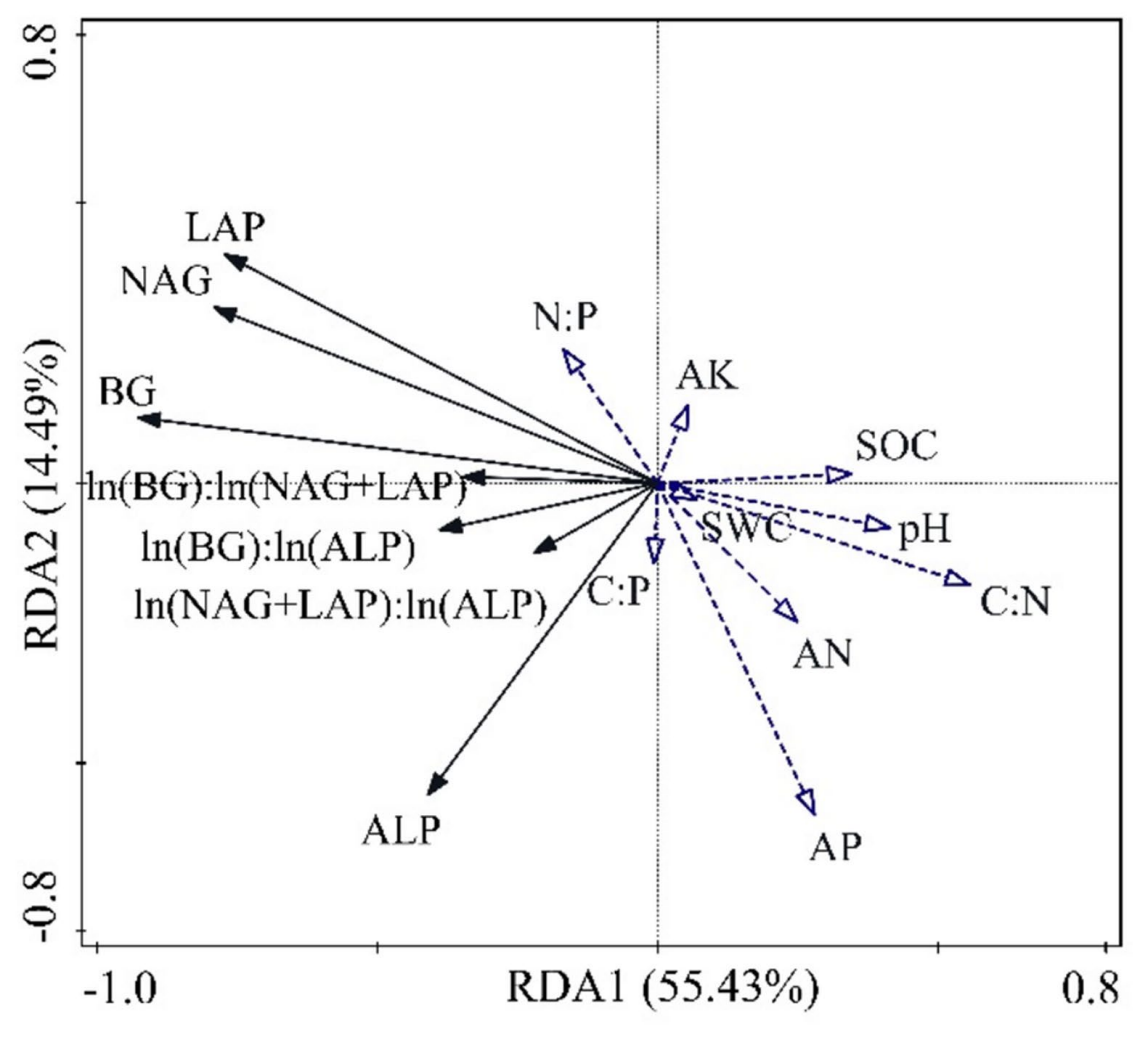
Redundancy analysis (RDA) of soil enzyme activity and its stoichiometric ratio with soil physicochemical factors.

Soil enzyme activity and its stoichiometric ratio were set as response variables, and redundancy analysis (RDA) was performed with soil physicochemical properties and C, N, and P stoichiometric ratios as explanatory variables. The results showed that the first axis explained 55.43% of the variables, and the second axis explained 14.49% of the variables (Figure 7). The first four ranking axes could cumulatively explain 71.01% of the variation in the relationship with soil enzyme activities and their stoichiometry (Table 1). The interpretation rates of C:P, N:P, C:N, AP, SOC, AK, and pH were 20.2, 18.2, 17.8, 8.3, 4.9, 1.0, and 0.6%, respectively. Among them, C:P (*p* = 0.002), N:P (*p* = 0.002), C:N (*p* = 0.004), AP (*p* = 0.006), and SOC (*p* = 0.044) had significant influences on enzyme activity and the stoichiometric ratio (Table 2). SOC and pH had a strong negative correlation with the soil enzyme stoichiometric ratio. LAP, NAG, and BG were negatively affected by soil C:N, AN, and pH.

**Table 1:** Parameter statistics of RDA analysis results.

Interpretation parameter	RDA1	RDA2	RDA3	RDA4
Eigenvalues	0.5543	0.1449	0.0098	0.0012
Cumulative variance explained/%	55.43	69.92	70.90	71.01
Pseudo-canonical correlations	0.9173	0.6977	0.5078	0.4567
Explained fitted variation (cumulative)	78.05	98.45	99.83	100.00
The sum of all eigenvalues	1.0000			

**Table 2:** Importance ranking and significance test results of interpretation of soil environment variables in *S. psammophila* sand barrier.

Environment factors	Explains/%	Contribution/%	Pseudo-*F*	*p*	Importance ranking
C:P	20.2	28.4	8.8	0.002	1
N:P	18.2	25.6	10.8	0.002	2
C:N	17.8	25.0	6.0	0.004	3
AP	8.3	11.7	5.8	0.006	4
SOC	4.9	6.8	3.8	0.044	5
AK	1.0	1.4	0.8	0.458	6
pH	0.6	0.8	0.4	0.668	7
SWC	0.1	0.2	<0.1	0.942	8
AN	<0.1	<0.1	<0.1	0.986	9

## 4 Discussion

### 4.1 Characteristics of physicochemical properties and enzyme activities of soils in *Salix psammophila* sand barriers of different years

The decay process of *S. psammophila* sand barrier bodies contributes to the nutrient cycling and nutrient use of desert ecosystems. The decomposition of sand barriers affects soil physicochemical properties and microbial community structure, thereby impacting soil enzymatic activity. Studies have shown that *S. psammophila* sand barriers can improve soil structure and soil nutrients (Zhang et al., 2020). The results of this study showed that the SOC and AN contents and enzyme activities were the lowest when the sand barrier had been set for 1 year. This is mainly because the study area was a quicksand environment before the setting of the sand barriers (Zhou et al., 2014), with low soil nutrients, microbial abundance, and enzymatic activity. The activity of ALP reflects the potential capacity of the soil to supply effective phosphorus and can be used to characterize the fertility of the soil (Nan et al., 2015). In this study, soil ALP reached its maximum value in the 6th year when the sand barrier was set. This may be due to the promotion of soil alkaline phosphatase activity by microbial communities (especially rare taxa) containing the alkaline phosphatase gene (*phoD*) (Xu et al., 2022). This was consistent with the findings of Liang et al. (2022) on the microbial biomass and soil enzyme activity of the soil of *S. psammophila* sand barriers. As time increases, the sand barrier begins to decay, and the breakage rate reaches its highest point at 5 years (Gong et al., 2011). This increases the contact area of the *S. psammophila* sand barrier with the soil, replenishes the SOC and TN content in the soil as the degree of decay increases, and provides a source of C and N for the survival of microorganisms. Therefore, the soil microorganisms around the mechanical sand barrier increase (Liang et al., 2021). However, as the sand barriers are continuously decomposed by microorganisms, the remaining available organic matter decreases and the release of nutrients into the soil gradually declines, leading to a decrease in soil microorganisms and a subsequent decrease in soil enzyme activity.

### 4.2 Influence of soil physicochemical properties on soil enzyme activity and its stoichiometric ratio

Changes in the physicochemical properties of the soil of the *S. psammophila* sand barriers at different setting years can have an important impact on soil enzyme activity and its stoichiometric ratio (Liang et al., 2022; Wang et al., 2019). Soil C, N, and P stoichiometry may regulate microbial nutrient metabolism limitation by influencing the microbial community composition and metabolism (microbial C use efficiency), thereby affecting nutrient cycling in desert ecosystems. In this paper, it was found that AP, SOC, and C, N, and P stoichiometry significantly affected the soil enzyme activities and their stoichiometric ratios. The study found that SOC was the main factor regulating changes in enzyme activity and enzyme stoichiometry ratios (Zhong et al., 2021). Taylor et al. (2002) also showed that there was a significant positive correlation between soil enzymes and SOC, further supporting the results of the present study. This is due to the ability of SOC to alter soil porosity, aeration, and soil aggregate structure, providing a major source of substrates for enzymatic reactions in soil (Zhang et al., 2021) and becoming the most complex system in the soil solid phase. SOC is the main environmental factor that directly limits soil microbial activity, amount, and community composition (He et al., 2017). Soil pH affects the decomposition and mineralization of soil organic matter, the aggregation and dispersion of colloids, and redox processes, and has a direct impact on the rate of participation of soil enzymes in biochemical reactions (Xie et al., 2018). Different types of enzymes have different optimal pH values. In this study, pH was found to be strongly negatively correlated with the activity of the four enzymes. This indicates that weakly alkaline soils may be an important factor in restraining enzyme activity.

LAP, NAG, and ALP reached their maximum points at 6 years, while BG reached a maximum at 5 years, which was consistent with the findings of Tian et al. (2019). The reason for this may be that the increase in moisture reduces the permeability and oxygen content of the soil, and enhanced anaerobic microbial activity leads to the accumulation of C, N, and P nutrients (Zhong et al., 2021; Verhoeven et al., 2006), and a corresponding increase in enzyme activity. The log-transformed ratio of soil C-, N-, and P-converting enzyme activities in this paper was found to be 0.8:1:1, which deviated from the 1:1:1 ratio of global ecosystems. This suggests relatively weak carbon-related metabolic activity in the soil, possibly resulting from inadequate organic carbon inputs or altered microbial community structure (Lillo et al., 2025).

### 4.3 Nutrient-limiting factors of *Salix psammophila* sand barriers in different setting years

The Vector L of soil enzyme stoichiometry first increased and then decreased with the years of the *S. psammophila* sand barrier setting. This demonstrated that the limitation of microorganisms by C first increased and then decreased with the increase in time. This may be because the *S. psammophila* sand barriers themselves are less decomposed in the early stages of their installation, and thus less available C enters the soil. However, in the later stage of sand barrier decay, the content of C in the soil increased, and the utilization of C by microorganisms increased, so the restriction was weakened. On the other hand, the degree of collapse and breakage of sand barriers reaches 70–80% after 5 years (Gong et al., 2011), increasing their contact area with the soil and promoting microbial decomposition. In addition, some graminoids with high C content appear late after the setting of the mechanical sand barrier. These Poaceae return their nutrients to the soil and reduce C restriction on microorganisms (Qiao et al., 2018).

The results of the study showed that soil microorganisms were limited by N in the initial stage of the *S. psammophila* sand barriers setting (<5 years) and by P in the later stage of the setting (>5 years). This may be because there was less plant growth on the early quicksand, which had limited access to replenish the soil with N, and after 5 years, due to the decomposition of the sand barriers, which replenished the N content of the soil. The decay of the *S. psammophila* sand barriers has improved the nature of the soil, promoting the restoration and growth of vegetation. Microorganisms have some adaptive mechanisms to cope with elemental limitations. Microorganisms upregulate high-affinity nitrogen transport systems, such as ammonium transporters (AmtB) and amino acid transporters, under nitrogen-limiting conditions to enhance uptake of scarce nitrogen sources in the environment (Liu et al., 2024). In addition, microbial up-regulation of high-affinity phosphate transport systems (e.g., PstSCAB) enhances uptake of inorganic phosphorus (Li et al., 2025). Over time, mechanical sand barriers promote the formation and development of biological soil crusts. It was found that biological crusts would form on the soil surface in the later stage of the *S. psammophila* sand barrier setting, thereby increasing N fixation (Song et al., 2009). Vitousek et al. (2002) found that in biological crusts in extreme environments, such as arid or semi-arid regions, N fixation will be more prominent, thus alleviating the N limitation of soil microorganisms. In addition, there may also be some biotic or abiotic factors that affect the nutrient cycling and limitations of the ecosystem in this area, and the coupling effect of the two needs to be further considered in an integrated manner.

## 5 Conclusion

With the increase in the setting years of *S. psammophila* sand barriers, the enzyme activities of BG, NAG, and LAP showed an increasing trend, followed by a decreasing trend. All three were mainly negatively affected by soil C:N, AN, and pH. RDA results showed that SOC and pH were strongly negatively correlated with the stoichiometric ratio of soil enzymes. Soil stoichiometric ratios were the main factors driving soil enzyme activities and their stoichiometry. Soil microorganisms were primarily N-limited during the first 5 years of the *S. psammophila* sand barrier setting and P-limited after 5 years. Five years is the threshold for the type of microbial nutrient limitation (N to P). Therefore, nitrogen fertilizer should be added to the soil appropriately for the first 5 years of the *S. psammophila* sand barriers, assisting in vegetation restoration, but after 5 years, phosphorus fertilizer should be added in small quantities to reduce elemental limitation of microorganisms and maintain the stability of the area.

## Data Availability

The original contributions presented in the study are included in the article/supplementary material, further inquiries can be directed to the corresponding author.
